# The Rise of the Nested Multicompartment Model in Synthetic Cell Research

**DOI:** 10.3389/fmolb.2021.750576

**Published:** 2021-09-03

**Authors:** Emiliano Altamura, Paola Albanese, Fabio Mavelli, Pasquale Stano

**Affiliations:** ^1^ Department of Chemistry, University of Bari Aldo Moro, Bari, Italy; ^2^ Department of Biological and Environmental Sciences and Technologies (DiSTeBA), University of Salento, Lecce, Italy

**Keywords:** synthetic cells, artificial cells, membrane proteins, vectorial chemistry, multicompartment, nested design, vesosomes, multivesicular vesicles

## Multicompartment Models in Synthetic Cell Research

The attractiveness of the “bottom-up” approach as a viable route for constructing cell-like systems (Luisi, 2002; Stano, 2019) is clearly evident by the ever increasing number of international projects and initiatives dedicated to this fascinating research (Supplementary Text S1). Such cell-like systems, simply called “synthetic cells” (SCs), “artificial cells” or “protocells” (although with slighly different nuances of meaning) are compartment-based systems (often, but not only, liposomes), capable of mimicking some aspects of cell behavior in a range of manners, and can be variously conceived in terms of materials, designs, and scopes. Even if current SCs are not alive, there is a recognized optimism among practicioners about the contribution of this research to basic and applied science, and there is the bet it will become one of the most important biotechnologies in the near future,—not resembling anything existing before—for example in nanomedicine (Leduc et al., 2007; Krinsky et al., 2018, Ding et al., 2018; Lussier et al., 2021). It seems a useful remark, just after mentioning nanomedicine, recalling that RNA-based anti-COVID vaccines—which actually are RNA-loaded lipid nanoparticles (Pilkington et al., 2021) have been actually developed thanks to decades of research on liposomes and other nanovectors. This suggests a highly relevant and pioneer role that current SC research might have on future ‘smart’ nanomedicine scenarios.

SC research is now well recognized within the “bottom-up” or “*in vitro*” or “cell-free” or “chemical” domains of synthetic biology. Pioneer research, however, dates back to the early 1990s, mainly referred to the construction of protocellular models of minimal complexity for origins-of-life studies (Walde et al., 1994; Oberholzer et al., 1995; Szostak et al., 2001). In that context a minimalist design is generally applied, which means the use of allegedly primitive materials (e.g., fatty acids, ribozymes, short peptides) (Chen et al., 2005), simple architectures (single, individual compartments), and essential functions (e.g., growth-division driven by basic physico-chemical events). Relevance is given to the verification of capabilities, constraints, and properties which might have ruled the primitive life-like dynamics of compartmentalized chemical systems.

**FIGURE 1:**
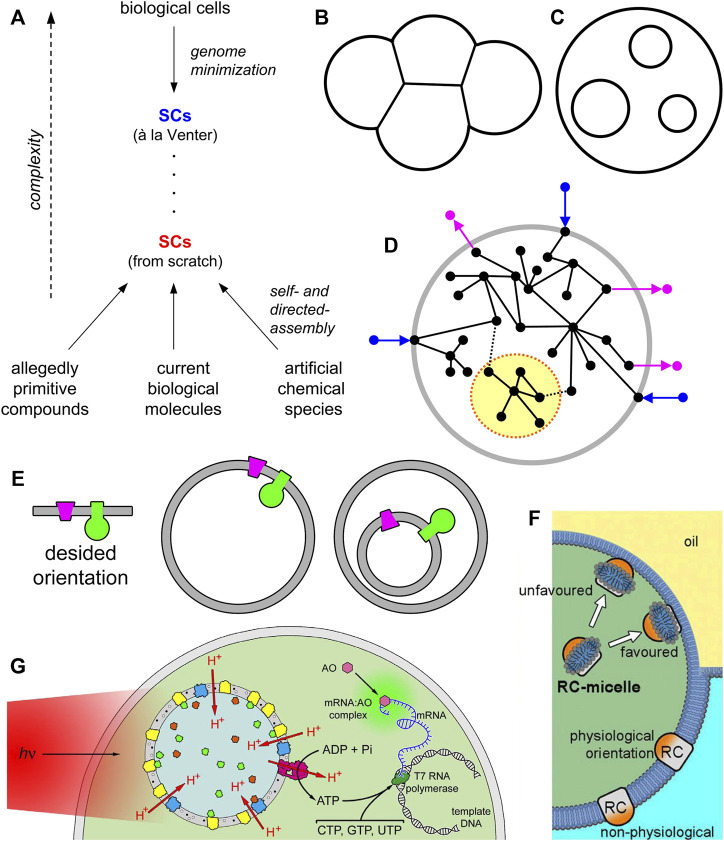
Synthetic cells (SCs) and the nested multicompartment design. **(A)** Schematic representation of SC research. SCs can be obtained from modern cells by a process of minimization, e.g., by designing, constructing, and insert a minimal genome in living cells. Such an approach has been pioneered by C. Venter (Gibson et al., 2010). The resulting minimal SCs are alive. Bottom-up SCs can be constructed from scratch, by employing different types of molecules (or mixtures of them). The construction grounds on self-assembly and directed-assembly processes. Up to date, bottom-up SCs are not alive and lie at a much lower complexity level when compared with SCs à la Venter. **(B)** “Flanked” or sidewise multicompartment SCs. **(C)** “Nested” multicompartment SCs, also known as multivesicular vesicles or vesosomes in liposome technology. **(D)** A pictorial representation of the concept of segregation (term borrowed by the dynamic systems theory) which conceptually corresponds to the idea of “module” in synthetic biology. In the dynamic chemical network that constitutes the SC (open to the environment) it is possible to indentify a sub-network whose relational links with the whole network are inferior in number, strength, and quality—because of physical or functional segregation. Note, however, that the “module” still interacts with the whole network, i.e., it is not relationally isolated from it. **(E)** A simplified cartoon showing that vectorial elements embedded in the membrane (or in general, in any interface) need to have a proper orientation. When such elements should be incorporated in single compartment or multicompartment SCs, their location (and mechanism of insertion) will correspondingly change dramatically. SC technology must allow the decoration of interface with vectorial elements in all possible configurations, at will. **(F)** Detergent-guided reconstitution of vectorial membrane proteins (MPs) in single compartment SCs from the inside. Image taken from Altamura et al. (2017) with the permission of the National Academy of Science United States ©2017. **(G)** Chromatophores from *Rhodobacter sphaeroides* can be employed as organellae-like particles inside giant vesicles, in order to construct SCs capable of producing ATP under illumination. Image taken from Altamura et al. (2021) with the permission of the National Academy of Science United States ©2021.

On the other hand, SC research has gradually expanded and has incorporated other approaches that enriched and favored its development. In particular, current studies include systems made of various materials, mainly modern biomacromolecules (following, then, a reconstitution philosophy), but also artificial ones (e.g., block copolymers, *ad hoc* designed reactive surfactants (Kurihara et al., 2011; Budin and Devaraj, 2012), etc.), and their various combinations, including allegedly primitive materials (Figure 1A). Solute-filled liposomes are largely—but not uniquely—employed for that scope. Indeed, the experimental approaches are inspired by the functional (and relational (Rosen, 1991)) roles of SC components rather than their material embodiment. In a sense, SCs are tools for investigating life as it was, as it is, and as it could be.

Interestingly, in addition to single-compartment design, representing SCs with an architecture of minimal complexity, multicompartment SCs can be constructed, leading to very interesting systems with peculiar features. The term “multicompartment” can refer to a “flanked” (sidewise, Figure 1B) or to a “nested” design (Figure 1C). In the first case, one refers to structures having flanked compartments, attached to each other, to generate 1D, 2D or 3D assemblies (or clusters), often considered models of tissues or multicellular systems (Carrara et al., 2012; Elani et al., 2013). In the second case, the SC architecture would resemble an eukaryotic cell, and the small compartments inside the large one mimic biological intracellular organellae (Bolinger et al., 2004; Elani et al., 2018). In the jargon of liposome technology, the latter structures are called “multivesicular vesicles” (MVVs) or “vesosomes” (Kisak et al., 2004; Giuliano et al., 2021). It should not escape from the attention that early studies on vesosomes were actually motivated by the need of constructing better drug delivery vehicles, for example by providing better protection of loaded drugs against degrading enzymes, or easier modular construction of drug cocktails with differential release (by tuning the membrane properties of individual internal compartments); for a detailed discussion, with examples, please refer to (Giuliano et al., 2021).

In this article we will briefly discuss the nested multicompartment architectures, to highlight their advantages, mainly when coupled to vectorial chemistry. As a casestudy we will highlight systems whereby membrane proteins (MPs) provide the necessary function to generate and/or exploit (electro)chemical gradients, for instance to produce ATP inside SCs.

## Nested Multicompartments: Construction, Modularization, and Vectorial Chemistry

### Construction

Peter Walde and collaborators have recently described in great detail the methods available for the construction of lipid vesicles containing other lipid vesicles, namely, nested multicompartment systems (Giuliano et al., 2021). However, most methods are quite specific, and can be used only under particular circumstances. A recent example that illustrates this principle is provided by the vesicle-to-sponge nanoparticle transition through the proliferation of membrane linking pores, a phenomenon that is finely controlled by amphiphilic composition of the membranes, and that leads to “spongosomes” (Angelova et al., 2019). In contrary, a significant step toward the construction of nested multicompartment SCs comes from those methods that lead to giant vesicles (GVs) starting from water-in-oil (w/o) droplets—the so-called droplet transfer method (Pautot et al., 2003; Dimova et al., 2020), or from water-in-oil-in-water (w/o/w) droplets. The inner aqueous solution that is employed in these methods is preliminarily provided with the small compartments suspended therein (Supplementary Figure S1). When GVs form, the small compartments will be found in the GV aqueous lumen. The entrapment efficiency is generally high; the procedure, then, is rather straigthforward. Moreover, when microfluidic devices are employed, the principles of operations are similar, and the process leads to nested multicompartment SCs in a very reproducible manner (e.g., (Haller et al., 2018)).

### Modularization

Any multicompartmentalized architecture implies a spatial and functional modularization of the whole system in sub-units. Let us consider a nested multicompartment SC as a wholeness, i.e., as a large chemical system made of several components. The compartmentalization of some components in separate sub-units (the internal vesicles), together with the limited (or absent) exchange of these components between the units, or between the units and the large compartment, *de facto* generates a modular system, characterized by a (partial or total) separation of some processes in space and in time. In the language of general systems theory (von Bertalanffy, 1968) this is called “segregation” and implies a (partial or total) decoupling between the processes pertaining to the system’s components (Figure 1D). As a result, the whole system (the SC) can be treated conceptually and practically as resulting from the sum of functions of its segregated components. This is clearly advantageous for making the construction easier, and reduces the possibility of unwanted interaction between components. Moreover, because this particular sort of modularization is based on physical segregation, it allows multiple milieu coexist in the SC. Modularization is a well known principle in synthetic biology, and although it has a reductionistic flavour, it is useful for the construction of systems with non trivial complexity. Note, however, that a total decoupling between the parts of a system conflicts with the concepts of wholeness, integration, and interactions which are prerequisites for displaying emergent properties.

### Vectorial Chemistry

The relevance of nested multicompartment design does not include only modularization, but also the possibility of generating a “vectorial” chemical processes which are unattainable in bulk (Harold, 1986), generally occurring at the interface between two sub-systems. The interface we refer to can be a lipid or polymer membrane (of vesicles), or even the interface of membraneless compartments. A prototypical example comes from vectorially operating membrane proteins (MPs) that translocate chemicals across an interface. Their operation generates a chemical gradient, and thus directly affecting the free energy—with a contribution proportional to log (C_in_/C_out_)—in the most fundamental physico-chemical form. As it is well known, living cells generate and exploit the so-called proton-motive force for producing ATP. The nested multicompartment design, when coupled to vectorial chemistry, efficiently leads to a “qualitative leap” directly into bioenergetics. Such a vectorial mechanism can reside either in the SC outmost boundary (the outer membrane), but also—and more conveniently—at the membrane of internal compartments in nested multicompartment SCs. In contrary to the first case, where the variability of environmental conditions would hamper its efficiency, mechanisms localized in the membrane of internal compartments work more efficiently as it is easier to keep stable the SC internal milieu. In other words, nested multicompartment design gains robustness for such kind of gradient-based mechanisms. A speculation about two additional effects possibly emerging from the nested multicompartment design is given in Supplementart Text S2, while Supplementary Text S3 is a brief commentary on the organization and complexity of multicompartment SCs.

## Membrane Proteins as Key Elements for Vectorial Chemistry in Single Compartment- and Nested Multicompartment-synthetic cells

One of the frontier research line in SC construction directly refers to bioenergetics, and deals with endogenous ATP production. Firstly, this is needed to feed processes necessary for complex SCs. Second, such a production would correspond to the emancipation from the current “windup toy” approaches based on endowing SCs, at time zero, with all required chemical energy to run, and the consequent ceasing when such supply runs out. Moreover, when the two cuncurrent processes of ATP production and usage are coupled, the system nicely constitutes a realization of out-of-equilibrium homeostasis (Pols et al., 2019).

The obvious idea is to engage SCs in an upstream phosphorylation process (ADP + Pi → ATP), operated by ATP synthase, and driven by an (electro)chemical proton gradient. The latter is vectorially generated by a membrane protein (MP) system capable of coupling redox or photoredox reactions with proton pumping. Such a goal, when realized by means of nested multicompartment SCs, combines the three concepts defined in *Nested Multicompartments: Construction, Modularization, and Vectorial Chemistry*.

Decorating the membranes with MP complexes—the ones that realize vectorial chemistry for ATP production, for instance, requires a precise orientation of all MPs involved in it, to avoid futile cycles. From the simplified drawing of Figure 1E, it is evident that SCs designed as single-compartment or as nested multicompartment require two opposite strategies for MP insertion. This means, in turn, that a complete control of this key process is required at any case: either insertion of pre-formed MP delivered with micelle (Jørgensen et al., 2017; Skrzypek et al., 2018), either insertion of nascent, ribosomally synthesized MP (Kuruma et al., 2009), and for any direction (from the inside or from the outside of the compartment).^1^

Systematic studies about vesicle “decoration” with vectorial MPs that include all above-mentioned possibilities are still lacking, although significant advancements have been recently reported for cell-free synthesis approaches (Sachse et al., 2014; Kuruma and Ueda, 2015; Niwa et al., 2015; Jacobs et al., 2019; Kruyer et al., 2021), demonstrating, for instance, MPs insertion in the lipid membrane can be guided bythe secYEG translocon (Matsubayashi et al., 2014).

In recent reports it has been shown how to reconstitute MPs with a proper orientation by treating GVs with MPs solubilized as micelles which were included in their aqueous lumen or in the external phase (Yanagisawa et al., 2011; Altamura et al., 2017), with high orientation, Figure 1F. These strategies can be adapted both for single-compartment and multi-compartment SC approaches.

The multi-compartment “nested” design leads to more complex SCs, but can be operatively simpler because the internal compartments can be prepared in advance (Biner et al., 2020), and later inserted in the larger one (the “host” vesicle). SCs designed as nested systems appear also more functional, as it is easier to control the SC internal milieu, providing optimal conditions for the operations of internalized small compartments. Modularization by sub-compartmentalization offers the additional advantage of segregating the elements present in the inner vesicle avoiding the mixing and possible noxious interactions with the other SC elements. The nested design is currently at the spotligth of SC research, as it has been employed by several relevant studies, not only for ATP production (Hindley et al., 2019; Belluati et al., 2020). As mentioned, this design is quite valuable for ATP production, being the internal compartments essentially a sort of organellae-like structures with a dedicated function. For example, the ATP-producing synthetic organellae (driven by irradiation) have been produced by detergent-driven reconstitution (Lee et al., 2018) or by directed assembly (Feng et al., 2016), or by direct insertion of the *in statu nascendi* cell-free synthesized membrane proteins (Berhanu et al., 2019). Alternatively, “prefabricated” and highly efficient organellae have been used, borrowing them from photosynthetic bacteria of the genus *Rhodobacter* (Altamura et al., 2021) (Figure 1G). Hybrid approaches (particles formed by thylakoid fragments of spinach plus lipids) have been also explored, but not inside SCs (Zheng et al., 2018).

## Concluding Remarks

In this Opinion article we have highlight a current trend in SCs research, namely the one moving from simple and isolated SCs to systems made of several compartments. “Flanked” (sidewise) or to a “nested” designs allow moving upward in complexity and favour the achievement of novel functions that will drive near-future directions in the field. MPs will be pivotal too. Think, for example, to G-Protein Coupled Receptors or other receptors as a way to access and exploit the sensorium toolbox also in SCs (May et al., 2013; Hamada et al., 2014; Gessesse et al., 2018). In particular, we have remarked that the nested multicompartment design ideally endows SCs with the energy-producing function and decisively contributes to next advancements.

## Footnotes

1 Stricter requirements are instead required when SCs are built aiming at autonomously producing their components (i.e., being “autopoietic”), MPs included. In that case, components must be produced from within, and thus the functionalization of membranes with highly oriented MPs becames a critical step. For example, see (Berhanu et al., 2019). For a succesful MP synthesis, insertion, and functioning, the SCs membrane should be made of a proper lipid mixture in order to 1) form stable vesicles, 2) do not interfere with transcription-translation, 3) host the MP in correct fold, 4) do not inhibit the MP functioning (Kuruma et al., 2009).

## References

[B49] Altamura E. Albanese P. Marotta R. Milano F. Fiore M. Trotta M. (2021). Chromatophores Efficiently Promote Light-Driven ATP Synthesis and DNA Transcription Inside Hybrid Multicompartment Artificial Cells. Proc. Natl. Acad. Sci. U.S.A. 118. 10.1073/pnas.2012170118 PMC7896284 33526592

[B1] Altamura E. Milano F. Tangorra R. R. Trotta M. Omar O. H. Stano P. (2017). Highly Oriented Photosynthetic Reaction Centers Generate a Proton Gradient in Synthetic Protocells. Proc. Natl. Acad. Sci. U.S.A. 114, 3837–3842. 10.1073/pnas.1617593114 28320948 PMC5393214

[B2] Angelova A. Angelov B. Garamus V. M. Drechsler M. (2019). A Vesicle-To-Sponge Transition via the Proliferation of Membrane-Linking Pores in ω-3 Polyunsaturated Fatty Acid-Containing Lipid Assemblies. J. Mol. Liquids 279, 518–523. 10.1016/j.molliq.2019.01.124

[B3] Belluati A. Thamboo S. Najer A. Maffeis V. Planta C. Craciun I. (2020). Multicompartment Polymer Vesicles with Artificial Organelles for Signal-Triggered Cascade Reactions Including Cytoskeleton Formation. Adv. Funct. Mater. 30, 2002949. 10.1002/adfm.202002949

[B4] Berhanu S. Ueda T. Kuruma Y. (2019). Artificial Photosynthetic Cell Producing Energy for Protein Synthesis. Nat. Commun. 10, 1325. 10.1038/s41467-019-09147-4 30902985 PMC6430821

[B5] Biner O. Fedor J. G. Yin Z. Hirst J. (2020). Bottom-Up Construction of a Minimal System for Cellular Respiration and Energy Regeneration. ACS Synth. Biol. 9, 1450–1459. 10.1021/acssynbio.0c00110 32383867 PMC7611821

[B6] Bolinger P.-Y. Stamou D. Vogel H. (2004). Integrated Nanoreactor Systems: Triggering the Release and Mixing of Compounds inside Single Vesicles. J. Am. Chem. Soc. 126, 8594–8595. 10.1021/ja049023u 15250679

[B7] Budin I. Devaraj N. K. (2012). Membrane Assembly Driven by a Biomimetic Coupling Reaction. J. Am. Chem. Soc. 134, 751–753. 10.1021/ja2076873 22239722 PMC3262119

[B8] Carrara P. Stano P. Luisi P. L. (2012). Giant Vesicles “Colonies”: A Model for Primitive Cell Communities. ChemBioChem 13, 1497–1502. 10.1002/cbic.201200133 22689306

[B9] Chen I. A. Salehi-Ashtiani K. Szostak J. W. (2005). RNA Catalysis in Model Protocell Vesicles. J. Am. Chem. Soc. 127, 13213–13219. 10.1021/ja051784p 16173749 PMC5072289

[B10] Dimova R. Stano P. Marques C. M. Walde P. (2020). “Preparation Methods for Giant Unilamellar Vesicles,” in The Giant Vesicle Book. Editors Dimova, R. Marques C. M. (Boca Raton, FL: Taylor & Francis Group), 3–20.

[B11] Ding Y. Contreras-Llano L. E. Morris E. Mao M. Tan C. (2018). Minimizing Context Dependency of Gene Networks Using Artificial Cells. ACS Appl. Mater. Inter. 10, 30137–30146. 10.1021/acsami.8b10029 30113814

[B12] Elani Y. Gee A. Law R. V. Ces O. (2013). Engineering Multi-Compartment Vesicle Networks. Chem. Sci. 4, 3332–3338. 10.1039/C3SC51164B

[B13] Elani Y. Trantidou T. Wylie D. Dekker L. Polizzi K. Law R. V. (2018). Constructing Vesicle-Based Artificial Cells with Embedded Living Cells as Organelle-like Modules. Sci. Rep. 8, 4564. 10.1038/s41598-018-22263-3 29540757 PMC5852042

[B14] Feng X. Jia Y. Cai P. Fei J. Li J. (2016). Coassembly of Photosystem II and ATPase as Artificial Chloroplast for Light-Driven ATP Synthesis. ACS Nano 10, 556–561. 10.1021/acsnano.5b05579 26615669

[B50] Gessesse B. Nagaike T. Nagata K. Shimizu Y. Ueda T. (2018). G-Protein Coupled Receptor Protein Synthesis on a Lipid Bilayer Using a Reconstituted Cell-Free Protein Synthesis System. Life 8, 54. 10.3390/life8040054 PMC6316570 30400226

[B15] Gibson D. G. Glass J. I. Lartigue C. Noskov V. N. Chuang R.-Y. Algire M. A. (2010). Creation of a Bacterial Cell Controlled by a Chemically Synthesized Genome. Science 329, 52–56. 10.1126/science.1190719 20488990

[B16] Giuliano C. B. Cvjetan N. Ayache J. Walde P. (2021). Multivesicular Vesicles: Preparation and Applications. ChemSystemsChem 3, e2000049. 10.1002/syst.202000049

[B17] Haller B. Göpfrich K. Schröter M. Janiesch J.-W. Platzman I. Spatz J. P. (2018). Charge-controlled Microfluidic Formation of Lipid-Based Single- and Multicompartment Systems. Lab. Chip 18, 2665–2674. 10.1039/C8LC00582F 30070293

[B18] Hamada S. Tabuchi M. Toyota T. Sakurai T. Hosoi T. Nomoto T. (2014). Giant Vesicles Functionally Expressing Membrane Receptors for an Insect Pheromone. Chem. Commun. (Camb.) 50, 2958–2961. 10.1039/c3cc48216b 24509495

[B19] Harold F. M. (1986). The Vital Force: A Study of Bioenergetics. New York: W. H. Freeman and Company.

[B20] Hindley J. W. Zheleva D. G. Elani Y. Charalambous K. Barter L. M. C. Booth P. J. (2019). Building a Synthetic Mechanosensitive Signaling Pathway in Compartmentalized Artificial Cells. PNAS 116, 16711–16716. 10.1073/pnas.1903500116 31371493 PMC6708380

[B21] Jacobs M. L. Boyd M. A. Kamat N. P. (2019). Diblock Copolymers Enhance Folding of a Mechanosensitive Membrane Protein during Cell-free Expression. PNAS 116, 4031–4036. 10.1073/pnas.1814775116 30760590 PMC6410776

[B22] Jørgensen I. L. Kemmer G. C. Pomorski T. G. (2017). Membrane Protein Reconstitution into Giant Unilamellar Vesicles: a Review on Current Techniques. Eur. Biophys. J. 46, 103–119. 10.1007/s00249-016-1155-9 27437691

[B23] Kisak E. T. Coldren B. Evans C. A. Boyer C. Zasadzinski J. A. (2004). The Vesosome - A Multicompartment Drug Delivery Vehicle. Curr. Med. Chem. 11, 199–219. 10.2174/0929867043456197 14754417

[B24] Krinsky N. Kaduri M. Zinger A. Shainsky-Roitman J. Goldfeder M. Benhar I. (2018). Synthetic Cells Synthesize Therapeutic Proteins inside Tumors. Adv. Healthc. Mater. 7, e1701163. 10.1002/adhm.201701163 29283226 PMC6684359

[B25] Kruyer N. S. Sugianto W. Tickman B. I. Alba Burbano D. Noireaux V. Carothers J. M. (2021). Membrane Augmented Cell-free Systems: A New Frontier in Biotechnology. ACS Synth. Biol. 10, 670–681. 10.1021/acssynbio.0c00625 33749249

[B26] Kurihara K. Tamura M. Shohda K.-I. Toyota T. Suzuki K. Sugawara T. (2011). Self-reproduction of Supramolecular Giant Vesicles Combined with the Amplification of Encapsulated DNA. Nat. Chem. 3, 775–781. 10.1038/nchem.1127 21941249

[B27] Kuruma Y. Ueda T. (2015). The PURE System for the Cell-free Synthesis of Membrane Proteins. Nat. Protoc. 10, 1328–1344. 10.1038/nprot.2015.082 26270393

[B28] Kuruma Y. Stano P. Ueda T. Luisi P. L. (2009). A Synthetic Biology Approach to the Construction of Membrane Proteins in Semi-synthetic Minimal Cells. Biochim. Biophys. Acta 1788, 567–574. 10.1016/j.bbamem.2008.10.017 19027713

[B29] Leduc P. R. Wong M. S. Ferreira P. M. Groff R. E. Haslinger K. Koonce M. P. (2007). Towards an *In Vivo* Biologically Inspired Nanofactory. Nat. Nanotechnol 2, 3–7. 10.1038/nnano.2006.180 18654192

[B30] Lee K. Y. Park S.-J. Lee K. A. Kim S.-H. Kim H. Meroz Y. (2018). Photosynthetic Artificial Organelles Sustain and Control ATP-dependent Reactions in a Protocellular System. Nat. Biotechnol. 36, 530–535. 10.1038/nbt.4140 29806849

[B31] Luisi P. L. (2002). Toward the Engineering of Minimal Living Cells. Anat. Rec. 268, 208–214. 10.1002/ar.10155 12382319

[B32] Lussier F. Staufer O. Platzman I. Spatz J. P. (2021). Can Bottom-Up Synthetic Biology Generate Advanced Drug-Delivery Systems? Trends Biotechnol. 39, 445–459. 10.1016/j.tibtech.2020.08.002 32912650

[B33] Matsubayashi H. Kuruma Y. Ueda T. (2014). *In Vitro* Synthesis of the *E. coli* Sec Translocon from DNA. Angew. Chem.-Int. Edit. 53, 7535–7538. 10.1002/anie.201403929 24894900

[B34] May S. Andreasson-Ochsner M. Fu Z. Low Y. X. Tan D. de Hoog H.-P. M. (2013). *In Vitro* Expressed GPCR Inserted in Polymersome Membranes for Ligand-Binding Studies. Angew. Chem. Int. Edition 52, 749–753. 10.1002/anie.201204645 23161746

[B35] Niwa T. Sasaki Y. Uemura E. Nakamura S. Akiyama M. Ando M. (2015). Comprehensive Study of Liposome-Assisted Synthesis of Membrane Proteins Using a Reconstituted Cell-free Translation System. Sci. Rep. 5, 18025. 10.1038/srep18025 26667602 PMC4678891

[B36] Oberholzer T. Wick R. Luisi P. L. Biebricher C. K. (1995). Enzymatic RNA Replication in Self-Reproducing Vesicles: an Approach to a Minimal Cell. Biochem. Biophys. Res. Commun. 207, 250–257. 10.1006/bbrc.1995.1180 7531971

[B37] Pautot S. Frisken B. J. Weitz D. A. (2003). Production of Unilamellar Vesicles Using an Inverted Emulsion. Langmuir 19, 2870–2879. 10.1021/la026100v

[B38] Pilkington E. H. Suys E. J. A. Trevaskis N. L. Wheatley A. K. Zukancic D. Algarni A. (2021). From Influenza to COVID-19: Lipid Nanoparticle mRNA Vaccines at the Frontiers of Infectious Diseases. Acta Biomater. 131 (21), 16–40. 10.1016/j.actbio.2021.06.023 34153512 PMC8272596

[B39] Pols T. Sikkema H. R. Gaastra B. F. Frallicciardi J. Śmigiel W. M. Singh S. (2019). A Synthetic Metabolic Network for Physicochemical Homeostasis. Nat. Commun. 10, 1–13. 10.1038/s41467-019-12287-2 31534136 PMC6751199

[B40] Rosen R. (1991). Life Itself. A Comprehensive Inquiry into the Nature, Origin, and Fabrication of Life. New York: Columbia University Press.

[B41] Sachse R. Dondapati S. K. Fenz S. F. Schmidt T. Kubick S. (2014). Membrane Protein Synthesis in Cell-free Systems: From Bio-Mimetic Systems to Bio-Membranes. FEBS Lett. 588, 2774–2781. 10.1016/j.febslet.2014.06.007 24931371

[B42] Skrzypek R. Iqbal S. Callaghan R. (2018). Methods of Reconstitution to Investigate Membrane Protein Function. Methods 147, 126–141. 10.1016/j.ymeth.2018.02.012 29454861

[B43] Stano P. (2019). Is Research on “Synthetic Cells” Moving to the Next Level? Life 9, 3. 10.3390/life9010003 PMC6463193 30587790

[B44] Szostak J. W. Bartel D. P. Luisi P. L. (2001). Synthesizing Life. Nature 409, 387–390. 10.1038/35053176 11201752

[B45] von Bertalanffy L. (1968). General System theoryFoundations, Development, Applications. London: Penguin.

[B46] Walde P. Goto A. Monnard P. Wessicken M. Luisi P. (1994). Oparin’s Reactions Revisited: Enzymic Synthesis of Poly(adenylic Acid) in Micelles and Self-Reproducing Vesicles. J. Am. Chem. Soc. 116, 7541–7547. 10.1021/ja00096a010

[B47] Yanagisawa M. Iwamoto M. Kato A. Yoshikawa K. Oiki S. (2011). Oriented Reconstitution of a Membrane Protein in a Giant Unilamellar Vesicle: Experimental Verification with the Potassium Channel KcsA. J. Am. Chem. Soc. 133, 11774–11779. 10.1021/ja2040859 21702488

[B48] Zheng D.-W. Xu L. Li C.-X. Dong X. Pan P. Zhang Q.-L. (2018). Photo-Powered Artificial Organelles for ATP Generation and Life-Sustainment. Adv. Mater. 30, e1805038. 10.1002/adma.201805038 30378187

