# Supplementary material for: The Rise of the Nested Multicompartment Model in Synthetic Cell Research
Source: Front Mol Biosci. 2021 Sep 3;8:750576. doi: 10.3389/fmolb.2021.750576 (PMC8446550; doi:10.3389/fmolb.2021.750576)
Supplement: Supplementary file 1 [file DataSheet1.pdf]

Supplementary Material

1 Supplementary Text

Text S1. For example the MaxSynBio at the Max Plank Institute in Germany, the BaSyC project and the SynCellEU initiative in the Netherlands, the Build-a-Cell initiative in the US, and the Fabricell group and the Bristol Centre for ProtoLife Research in UK, just to mention some of them. There is also a quite consolidated research community in Japan (CREST-PRESTO funding program by the JST, the Japanese Society for Cell Synthesis Research). Early projects were funded by the EU-FP6, such as PACE (Programmable Artificial Cell Evolution) and SYNTHCELLS.

Text S2. The presence of reactions localized in internal compartments can also lead to a second vectorial effect. Directed molecular flows (inward and outward) result as direct consequence of production/consumption of chemicals by localized reactions. In contrast to “scalar” reactions (homogeneously distributed in bulk), these reaction-diffusion subsystems can lead to higher degree of dynamical organization because they couple reaction and diffusion. Examples of emergent behavior in reactions whose dynamics depend both on reaction and diffusion rate are oscillatory reactions, wave-like phenomena as well as other self-organized patterns. Furthermore, the surface of the internal compartments, just because their existence, adds to the overall surface/volume ratio, further favouring (or better, being available for) the spatial ordering of molecules and processes. Surfaces deprive adsorbed molecules of degrees of freedom, and this is itself an organizing principle that could lead, for example, to supramolecular hyperstructures.

Text S3. Following the spirit outlined in a recent review[1], one can ask whether it is possible to rank different types of SCs based on complexity. It resulted that complexity can be related both the structure and function. In the flanked compartment design, the system complexity behaves generally additively, as each compartment lies at the same ’hierarchical’ level, (differences can arise from topological relations, for example when compartments at the edges of the 1D, 2D or 3D ensembles are considered; or, in more elaborate desings, when a differentiation among the compartments is created before the assemblage). In the nested multicompartment design, the internal compartments lie at a different hierarchical level than the larger compartment they are enclosed in, automatically generating upward/downward trains of causation. To confirm this basic intuition, we then call for a formal analysis of how different SC designs rank in a complexity scale, possibly contributing to some missing aspects of theoretical discussions on SCs.

---

1 Stano, P. (2019). Is Research on “Synthetic Cells” Moving to the Next Level? *Life* 9, 3. doi:10.3390/life9010003.

2 Supplementary Figures

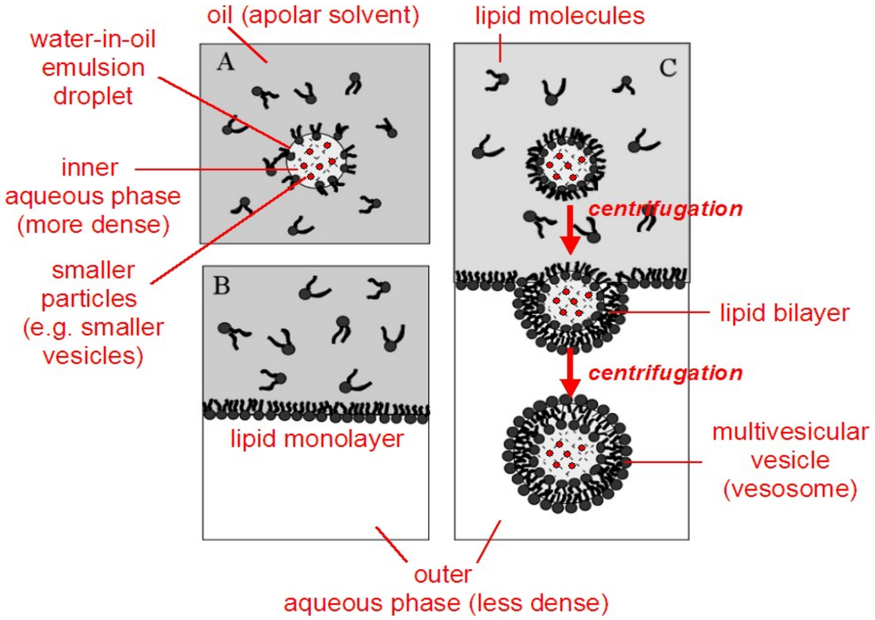

Supplementary Figure 1. Essentials of the "droplet transfer method" for the production of nested multicompartment SCs. The figure is taken and adapted, with permission of the American Chemical Society, from Pautot, S., Frisken, B. J., and Weitz, D. A. (2003). Production of unilamellar vesicles using an inverted emulsion. Langmuir 19, 2870–2879. doi:10.1021/la026100v, ©2003. The original figure is in gray tones; all indications in red have been added to adapt the figure to the present discussion. The drawing is not to scale. An inner aqueous solution (made more dense by the presence of 0.2 M sucrose), containing all the solutes that must be encapsulated in the giant unilamellar vesicles, is firstly prepared. For the construction of nested multicompartment SCs, the smaller particles that need to be encapsulated in the larger host vesicle, are present in the inner aqueous phase. The inner aqueous phase is emulsified in a phospholipid-containing mineral oil solution ("oil"). The resulting chromatophore-containing water-in-oil emulsion droplets – which are stabilized by a phospholipid monolayer – are then transferred, by centrifugation, to an underlying outer aqueous solution (made less dense – yet isotonic with the inner solution – by the presence of 0.2 M glucose). When the water-in-oil droplets cross the oil/aqueous interface, they become covered by a second phospholipid monolayer, and generate multivesicular vesicles (vesosomes) based on the giant vesicle structure.
